# Densely Functionalized 2-Methylideneazetidines: Evaluation as Antibacterials

**DOI:** 10.3390/molecules26133891

**Published:** 2021-06-25

**Authors:** Giovanni Petrillo, Cinzia Tavani, Lara Bianchi, Alice Benzi, Maria Maddalena Cavalluzzi, Lara Salvagno, Laura Quintieri, Annalisa De Palma, Leonardo Caputo, Antonio Rosato, Giovanni Lentini

**Affiliations:** 1 Department of Chemistry and Industrial Chemistry, University of Genoa, Via Dodecaneso 31, I-16146 Genoa, Italy; cinzia.tavani@unige.it (C.T.); lara.bianchi@unige.it (L.B.); alice.benzi@edu.unige.it (A.B.); 2 Department of Pharmacy–Pharmaceutical Sciences, University of Bari Aldo Moro, Via E. Orabona n. 4, 70126 Bari, Italy; mariamaddalena.cavalluzzi@uniba.it (M.M.C.); lara.salvagno89@gmail.com (L.S.); antonio.rosato@uniba.it (A.R.); giovanni.lentini@uniba.it (G.L.); 3 Institute of Sciences of Food Production (CNR-ISPA) National Council of Research, Via G. Amendola, 122/O, 70126 Bari, Italy; laura.quintieri@ispa.cnr.it (L.Q.); leonardo.caputo@ispa.cnr.it (L.C.); 4 Department of Biosciences, Biotechnologies, and Biopharmaceutics, University of Bari Aldo Moro, Via E. Orabona 4, 70126 Bari, Italy; annalisa.depalma@uniba.it

**Keywords:** antibiotics, drug resistance, gut microbiota, vinylogy

## Abstract

Twenty-two novel, variously substituted nitroazetidines were designed as both sulfonamide and urethane vinylogs possibly endowed with antimicrobial activity. The compounds under study were obtained following a general procedure recently developed, starting from 4-nitropentadienoates deriving from a common β-nitrothiophenic precursor. While being devoid of any activity against fungi and Gram-negative bacteria, most of the title compounds performed as potent antibacterial agents on Gram-positive bacteria (*E. faecalis* and three strains of *S. aureus*), with the most potent congener being the 1-(4-chlorobenzyl)-3-nitro-4-(*p*-tolyl)azetidine **22**, which displayed potency close to that of norfloxacin, the reference antibiotic (minimum inhibitory concentration values 4 and 1–2 μg/mL, respectively). Since **22** combines a relatively efficient activity against Gram-positive bacteria and a cytotoxicity on eucharyotic cells only at 4-times higher concentrations (inhibiting concentration on 50% of the cultured eukaryotic cells: 36 ± 10 μM, MIC: 8.6 μM), it may be considered as a promising hit compound for the development of a new series of antibacterials selectively active on Gram-positive pathogens. The relatively concise synthetic route described herein, based on widely available starting materials, could feed further structure–activity relationship studies, thus allowing for the fine investigation and optimization of the toxico-pharmacological profile.

## 1. Introduction

Eighty years ago, the modern era of antimicrobial therapy started. Since then, hundreds of antibacterials have been successfully used as clinically valuable agents to fight one of the main threats to human health, substantially contributing to the prolongation of life expectancy in the past century. Unfortunately, the widespread use (and misuse) of the ‘miracle drugs’ [1] has caused the emergence of antibacterial-resistant microorganisms, most of which display pathogenic potential. In particular, six multidrug-resistant pathogens (MDR: *Enterococcus faecium, Staphylococcus aureus, Klebsiella pneumoniae, Acinetobacter baumannii, Pseudomonas aeruginosa*, and *Enterobacter* spp.) were named ESKAPE pathogens due to their ability to ‘escape’ the biocidal action of antimicrobials [2]; such pathogens are responsible for the majority of nosocomial infections associated with higher rates of morbidity and mortality. The World Health Organization (WHO) has also recently classified ESKAPE pathogens in a list of 12 pathogens grouped as critical, high and medium priority, according to the urgency for new antibiotics [3]. The critical-priority list includes carbapenem resistant *A. baumannii*, P. aeruginosa, *K. pneumoniae*, and *Enterobacter* spp.; whereas vancomycin-resistant *E. faecium (VRE)* and methicillin and vancomycin-resistant *S. aureus* (MRSA and VRSA) are listed in the high-priority group. Besides the need to improve the surveillance protocols of antimicrobial resistance, it is urgent to find alternative strategies to cure infections, especially those caused by ESKAPE pathogens [4].

**Figure 1:**
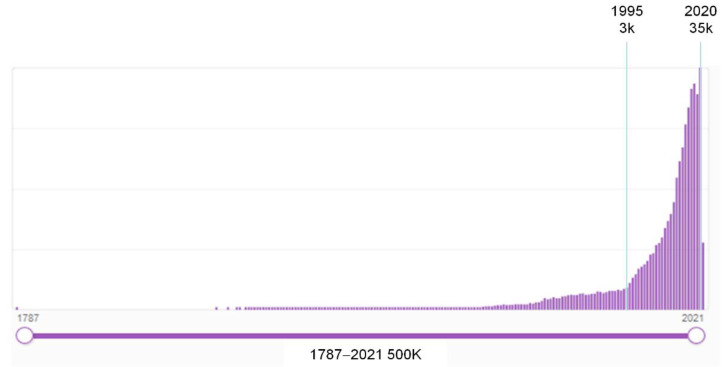
Number of answers per year from SciFinder ^n^ to the query ‘antibacterials’ [5].

The compelling need for new efficient drugs to support antimicrobial chemotherapy is demonstrated by the ever-increasing number of research groups focusing on this issue, mirrored, in turn, by the increase displayed by the number of relevant publications in the last five lustra (Figure 1).

Despite the effort, success in the development of antibacterial agents has been limited since several aspects (i.e., potency, pharmacokinetics and safety) have to be optimized when trying to rationally design new antibacterials, while the corresponding parameters are often contrasting [6]. The phenotypic screening of small libraries, i.e., whole bacteria treated with small libraries of molecules, seems to be a relatively less demanding approach to finding hit compounds [7].

**Figure 2:**
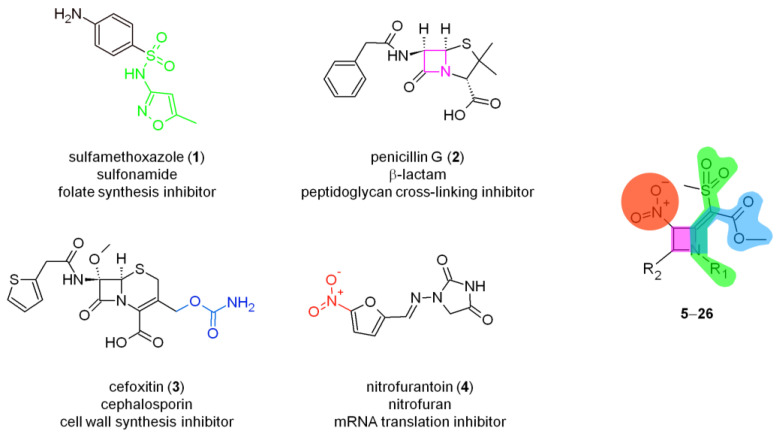
Structures of commonly used antibacterial agents characterized by a sulfonamide group (**1**), an azetidine core (**2**), a carbamate group (**3**) or a nitro substituent (**4**), and the general structure for the designed compounds **5**–**26**. Antibacterial classification and mechanism of action were included.

When considering the most successful, commonly used classes of antibacterials, we noticed the frequent presence of some structural elements such as the azetidine core and sulphonamide, carbamate, and nitro groups (Figure 2). Thus, we speculated that gathering all of the above motifs in the same structure would confer antibacterial activity to the so-designed small molecule congeneric series (**5**–**26**). The general structure reported in Figure 2 could be intended as the result of a scaffold-hopping strategy [8] applied to both sulfonamides and β-lactams. For the vinylogy principle [9], the methylsulfonyl group (green) should behave as a sulphonamide and the methoxycarbonyl group (blue) should be roughly endowed with the same electron density of a carbamate. As a matter of fact, a series of azetidine-containing compounds showed antibacterial, antifungal and antitubercular activities in vitro depending on the substituent on the 4-aryl group, according to the sequence: NO_2_ > Cl > Br > OH >> OCH_3_ > CH_3_ [10]. Conversely, among six azetidines bearing different phenyl/heterocyclic moieties, the one bearing the indole moiety displayed the highest antibacterial activity, probably due the resemblance with the β-lactam ring [11].

Considering that the introduction of a nitro group generally improves the antibacterial activity, we felt encouraged to prepare the nitroazetidines **5**–**26** and to test them as antimicrobials.

## 2. Results and Discussion

**Scheme 1:**
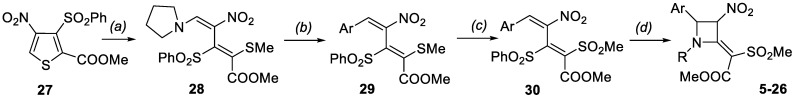
Synthesis of azetidines **5**–**26** starting from the common precursor **27**. (**a**): pyrrolidine, AgNO_3_, EtOH, then MeI; (**b**): ArMgX, THF, then acidic quenching; (**c**): *m*-CPBA, DCM; (**d**): RNH_2_, DCM.

The title compounds were obtained according to the procedure reported in Scheme 1 [12], which, starting from nitropentadienoates in turn derived from a common nitrothiophenic precursor (Scheme 1), resulted in general efficiency and applicability. Briefly, the ring-opening of the thiophenic precursor **27** by pyrrolidine produces the nitroenamine **28**, from which the aryl pentadienoates **29** and **30** can be conveniently obtained. Treating **30** with primary amines, the corresponding aza-Michael adducts are generated, which cyclize to the nitroazetidines of interest.

The title compounds were tested as antimicrobials against selected pathogens belonging to the ATCC collection; they included Gram-positive bacteria *Staphylococcus aureus* (ATCC 29213, 6538 P, and 6538), and *Enterococcus faecalis* (29212), Gram-negative bacteria *Pseudomonas aeruginosa* (ATCC 27853), *Escherichia coli* (ATCC 25922), and *Klebsiella pneumoniae* (ATCC 13883), and fungi *Candida albicans* (ATCC 10231), *Candida albicans* (ATCC 90028), *Candida glabrata* (ATCC 15126), *Candida tropicalis* (ATCC 750), *Candida kefyr* (ATCC 204093), *Candida krusei* (ATCC 6258). Clinical isolates (*Candida albicans* A18, *Candida albicans* 810, *Candida krusei* 31A29, *Candida parapsilosis* 11A13, *Candida parapsilosis* 1A1) were also included. The activity was measured as the minimum inhibitory concentration (MIC) that is the concentration (μg/mL, γ) that prevents any visible bacterial growth. All the compounds tested were inactive against both Gram-negative bacteria and fungi (MIC > 512 γ) but displayed significant activity against Gram-positive bacteria (Table 1). These results were in accordance with the spectrum of activity of β-lactams antibiotics; these latter, indeed, were able to induce cell wall destabilization after diffusing through the bacterial cell wall. In contrast to Gram-positive bacteria, Gram-negative ones have an additional lipopolysaccharide layer that decreases antibiotic penetration.

In general, the most active compounds were the benzyl *N*-derivatives **14**–**26**, with interesting differences based on the number (*n* = 0–2) and position of chlorine atoms on the phenyl ring. In particular, the *o* < *m* < *p* trend of activity was observed for the *N*-chlorobenzyl analogues **16**–**18** and **20**–**22**, thus indicating the *para* position as the preferred one for the antibacterial activity. When a second chlorine atom was introduced in the most potent *p*-chloro substituted compounds (**18** and **22**) to obtain the 3,4-dichlorobenzyl analogues **19** and **23**, respectively, the activity was mostly affected in the *p*-tolyl series with compound **23** showing 2–8-fold higher MIC values (i.e., lower activity) against the four bacterial species tested. On the contrary, the absence of a chlorine atom led to a total loss of activity against *E. faecalis* in the R^1^ = benzyl, R^2^ = *p*-tolyl azetidine derivatives (**14**, **20**–**23**), with the MIC value of **14** being > 512 μg/mL; no dramatic effects were observed in the 1-benzyl-2-thienyl series (**15**–**19**). Furthermore, a peculiar behavior was observed in *S. aureus* ATCC 6538 in both *p*-tolyl and 2-thienyl azetidine series, since a higher activity was reached when the chlorine atom is absent rather than present in the *ortho* and *ortho*/*meta* positions, respectively. In general, among the benzyl analogues **14**–**23**, slightly higher activity on all tested strains was observed when a *p*-tolyl moiety, rather than 2-thienyl, was linked at the 2-position of the azetidine core, regardless of the substituent in the 1-position. The alkyl or cycloalkyl substitution (**5**–**12**) was not well tolerated, leading to a loss of potency (MIC > 512 μg/mL), except for compound **12** showing the same MIC value as **16** against two of the three *S. aureus* strains (ATCC 29,213 and ATCC 6538P). Unlike the latter strains, **16** was completely inactive against *E. faecalis*. In general, the observed antibacterial activity was not strictly correlated with lipophilicity (cLog *P*). However, a roughly parabolic trend might be seen, with optimal cLog *p* values being within the 1–3 range. The highest activity was obtained for compound **22**, whose MIC value (4 μg/mL) was only 2-fold higher than the reference compound norfloxacin against the *E. faecalis*, an important cause of life-threatening multidrug-resistant bacterial nosocomial infections.

**Figure 3:**
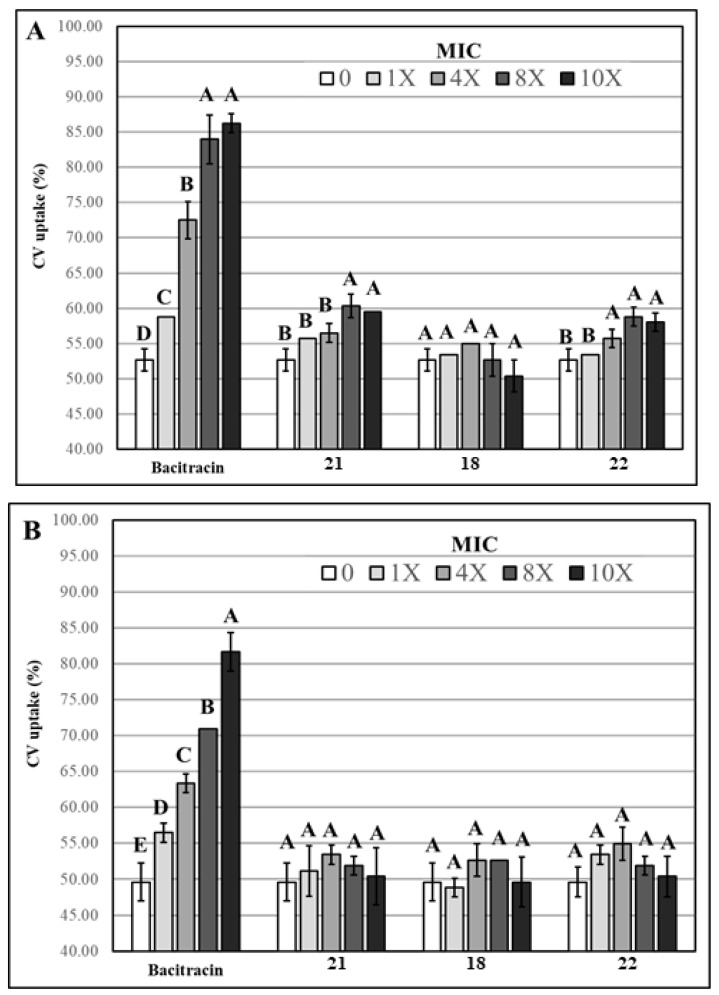
Crystal Violet (CV) uptake assay. The percentage (%) of CV uptake from *S. aureus* ATCC 6538P (**A**) and *E. faecalis* ATCC 29,212 cells (**B**), either untreated (0) or treated with bacitracin, **21**, **18** and **22** at 1X, 4X, 8X, 10X MIC for 1 h at 37 °C. Bars represent the means ± standard deviation. Different superscript letters A–E indicate average values significantly different (*p* < 0.05) according to post hoc Tukey test.

Looking for a possible mechanism behind the observed antibacterial activity, a preliminary study was carried out to see if our compounds might act via bacterial cytoplasmic membrane disruption. Increases in cell envelope permeability after treatment with the nitroazetidines **18**, **21** and **22** were analyzed by the crystal violet (CV) assay, through evaluation of the percentage of its uptake after incubation with the antimicrobial agent as previously reported [13,14]. The increase in CV uptake was generally associated with the membrane leakage due to a permeability change [13,14,15]. The use of the antibiotic bacitracin, well known to inhibit cell wall biosynthesis [16], as reference for the assay, confirmed the increase of CV uptake also when the cell wall integrity was compromised. Both *S. aureus* ATCC 6538 P and *E. faecalis* ATCC 29,212 strains were sensitive to bacitracin as reported by the BacDive database [17]. As reported in Figure 3, untreated cells registered ca. 51% uptake. As expected, a significant dose-response effect for bacitracin on CV uptake was registered for both strains (*p* < 0.05; ranging from 57.63 to 83.97%, with concentration values from 0.1 mg/mL to 1 mg/mL). A slight but significant increase in CV uptake values (56.34%, on average) was registered only for *S. aureus* cells treated with **18** and **21** at concentrations 8 and 10-fold higher than their MIC. No differences were instead registered for *E. faecalis* regardless of the assayed compounds. Although a slight membrane permeability was registered for **18** and **21**, the results exclude that the antimicrobial activity registered against *S. aureus* and *E. faecalis* might be caused by a cell envelope destabilization.

Although the β-lactam mechanism of action is due to the covalent interaction with the penicillin-binding proteins (PBPs), involved in peptidoglycan biosynthesis, structural changes (*N*-alkylation, ring opening and type of substituents at the C3 and C4 positions) can determine additional modes of action (modifications of the bacterial cell uptake, membrane permeability or inhibition of fatty acid synthesis) as suggested by Revell et al., 2007 [18]. Recently, azetidines endowed with bactericidal activity against *Mycobacterium tuberculosis* were also synthesized; mode of action and target deconvolution studies suggest that these compounds inhibit mycobacterial growth by interfering with cell envelope biogenesis, specifically late-stage mycolic acid biosynthesis. In addition, these compounds did not give rise to emerging specific resistance in mycobacterial model organisms; the inability to generate resistant mutants against the most active compounds suggests that this series of compounds may elicit pleiotropic activity or have non-specific modes of action, or have non-protein target(s) [19].

Since the structures of nitroazetidines **5**–**26** include an allegedly electron-poor ylidene C=C bond, it might be hypothesized that they may act as covalent modifiers of transpeptidase-like β-lactams. This would be in agreement with the observed high selectivity towards Gram-positive bacteria. To check this hypothesis, we run computational investigations on the geometrical and electronic properties of compound **22**, to be compared with the corresponding ones in penicillin G (**2**), chosen as the most typical antibacterial β-lactam. The relationships between β-lactam stereoelectronic features and the biological activity are well established and are qualitatively reported in the fourth column of Table 2 [20].

**Figure 4:**
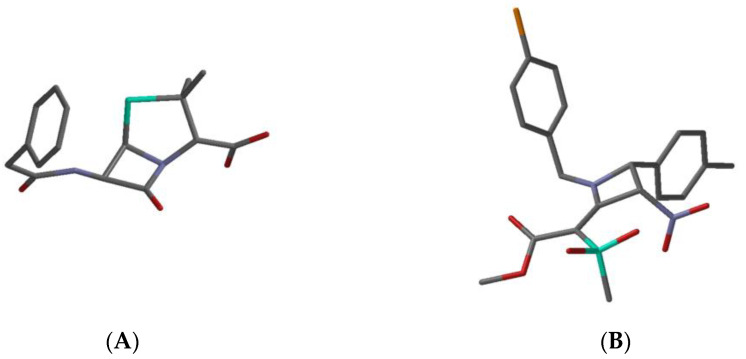

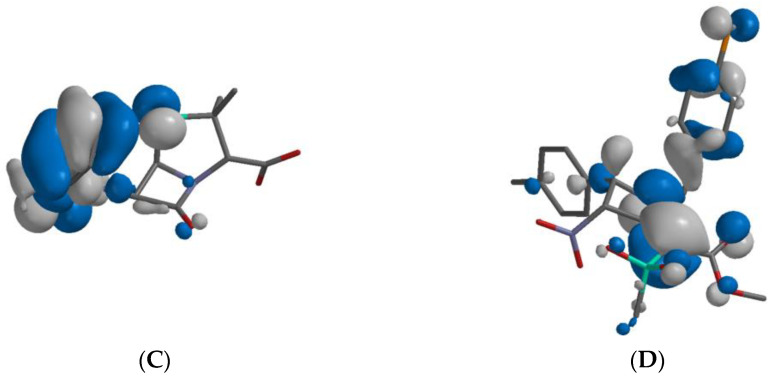
Geometry optimized models (DFT ῶB97X-D/6-31G*) of the structures of penicillin G (**A**) and nitroazetidine **22** (**B**) and corresponding HOMO density maps ((**C**) and (**D**), respectively).

The study was run at the DFT ῶ B97X-D/6-31G* level (gaseous phase) on one of the two most stable conformations of **2**, namely the folded one (Figure 4A) [21,22]. The same analysis was performed on the global minimum conformer of **22**, obtained through our previously developed conformational analysis procedure (Figure 4B). [23,24].

Most of the properties reported in Table 2 indicate that the azetidine ring in our compounds might hardly be considered as a potential electrophilic reagent like β-lactams, with the only electrophilicity index ῶ winking to that direction. On the contrary, the distribution found in the HOMO density maps (Figure 4C,D) seems to assign a local nucleophilic potential to the ylidene C=C bond. What is discussed above seems to indicate that compound **22** should not act as a covalent modifier of transpeptidase even though this conclusion does deserve further theoretical and experimental studies. As an early observation supporting the relatively low electrophilic potential of our compounds, we have observed high stability of azetidine **5**–**26** under harsh alkaline conditions.

Since aliphatic nitro-derivatives are expected to be potentially toxic to eukaryotic cells [25], the most efficient [26] antibacterial congeners **18**, **21** and **22** underwent preliminary acute toxicity profile evaluation through the platform Percepta (ACD/labs 2020.1.0). The software indicated compounds **18**, **21** and **22** as agents potentially able to cause hepatic damage (median LD_50_: 120 mg/Kg for 11 aliphatic nitro-compounds in the training database). Compounds **18**, **21** and **22** were predicted as equally moderately hazardous since they share the same LD_50_ values in each animal model:

mouse (ip): 190 mg/kg
mouse (po): 2300 mg/kg
mouse (iv): 56 mg/kg
mouse (sc): 46 mg/kg
rat (ip): 51 mg/kg
rat (po): 2400 mg/kg.

The most efficient congener **22** was then tested for cytotoxicity on eukaryotic cells (hepatocarcinoma cells, HepG2) [27] and displayed detrimental effects only at 4-times higher concentrations than its MIC (inhibiting concentration on 50% of the cultured eukaryotic cells: 36 ± 10 μM).

## 3. Materials and Methods

### 3.1. Chemistry

Materials and methods: ^1^H NMR and ^13^C NMR spectra were recorded with a Varian Mercury 300 Plus spectrometer, at 300 and 75 MHz, respectively, or with a Jeol JNM-ECZ400R, at 400 and 101 MHz, respectively; chemical shifts (TMS as internal reference) are reported as δ values (ppm). Signals are designated as follows: s, singlet; d, doublet; dd, doublet of doublets; t, triplet; q, quartet; m, multiplet. Gas chromatography–mass spectrometry (GC-MS) was performed on a HP 5890/5971 (EI 70 eV) system equipped with a HP-1 MS capillary column (12 m × 0.2 mm i.d × 0.33 μm). High-resolution mass spectra (HRMS) were obtained with an Agilent MSD TOF mass spectrometer, and recorded in positive ion mode with an electrospray (ESI) source. Melting points were determined with a Büchi 535 apparatus and are uncorrected. IR spectra were recorded on a Perkin Elmer Spectrum 65 FT-IR and wave numbers are reported in cm^−1^. Petroleum ether and light petroleum refer to the fractions with bp 40–60 °C and 80–100 °C, respectively. Silica gel 230–400 mesh was used for column chromatography, all solvents being distilled before use. TLC analysis were performed on commercially prepared 60 F254 silica gel plates and visualized by UV irradiation (eluent: petroleum ether–ethyl acetate). Tetrahydrofuran (THF) dry was commercial. All other commercially available reagents were used as received.

The starting nitrothiophene **27**, the 5-pyrrolidin-1-ylpentadienoate **28**, all the 2-methylthio- and 2-methylsolfonyl-5-arylpentadienoates **29** and **30** have been already described [12,30]. Two new substrates, the 2-methylthio and 2-methylsolfonyl-5-cyclohexylpentadienoates (**29e** and **30e**, respectively), were prepared following the same procedure, and their characterization is reported below. Synthesis and characterization of azetidines **5**–**9**, **12**–**14** have been already reported; [12] for the new azetidines **10**, **11**, **15**–**26**, obtained following the same procedure, the characterization is reported below.

Typical procedure for the cyclization of methyl 2-methylsolfonyl-4-nitro-3-phenylsolfonylpentadienoates to azetidines:

In a flask, the relevant nitropentadienoate (0.15 mmol) was dissolved in DCM (2.8 mL) and the necessary amine (3.3 mol equiv.) was added by syringe under magnetic stirring. The reaction was followed by TLC and at the end, the solvent evaporated under reduced pressure. The crude was purified by chromatography on a silica gel column using petroleum ether/ethyl acetate mixtures as eluent to obtain the title compounds, as pure cis/trans diastereomeric mixtures (each diastereoisomer as racemic mixture), interconverting on time, to the final equilibrium ratios.

Characterization of new compounds (**29e**, **30e**, **10**, **11**, **15**–**26**):

*Methyl (2E,4E)-5-cyclohexyl-2-(methylthio)-4-nitro-3-(phenylsulfonyl)penta-2,4-dienoate* (**29e**). White solid. M.p. 192.5–193.2 °C (Ethanol). ^1^H NMR (CDCl_3_, 300 MHz) δ 0.99–1.41 (4H, m), 1.57–1.83 (6H, m), 2.10–2.29 (1H, m), 2.34 (3H, s), 4.06 (3H, s), 7.36 (1H, d, *J* 11.6 Hz), 7.42–7.56 (2H, m), 7.55–7.65 (1H, m), 7.83–7.95 (2H, m). ^13^C NMR (CDCl_3_, 101 MHz) δ 14.88, 24.77, 24.95, 25.62, 30.85, 31.71, 39.04, 53.57, 123.15, 128.70, 129.33, 134.18, 138.79, 139.44, 151.97, 155.27, 163.21. HRMS (ESI) *m/z* calcd [M + H]^+^ C_19_H_24_NO_6_S_2_^+^ 426.1040, found 426.1042.

*Methyl (2E,4E)-5-cyclohexyl-2-(methylsulfonyl)-4-nitro-3-(phenylsulfonyl)penta-2,4-dienoate* (**30e**). White solid. ^1^H NMR (CDCl_3_, 300 MHz) δ 1.01–1.37 (4H, m), 1.74 (6H, d, *J* 14.2 Hz), 2.22–2.40 (1H, m), 3.18 (3H, s), 4.09 (3H, s), 7.33 (1H, d, *J* 11.9 Hz), 7.56 (2H, t, *J* 7.9 Hz), 7.66–7.78 (1H, m), 7.81–7.93 (2H, m). ^13^C NMR (CDCl_3_, 101 MHz) δ 24.67, 24.88, 25.59, 30.56, 30.68, 39.15, 42.19, 54.77, 129.78, 130.02, 135.80, 135.86, 136.31, 141.03, 145.68, 151.83, 162.10. HRMS (ESI) *m/z* [M + H]^+^ 458.0935 (calcd for C_19_H_24_NO_8_S_2_^+^, 458.0938).

*Methyl (E)-2-(1-butyl-4-(4-(methylsulfonyl)phenyl)-3-nitroazetidin-2-ylidene)-2-(methylsulfonyl) acetate* (**10**). Yellow solid. ^1^H NMR (CDCl_3_, 300 MHz) δ 0.80–0.98 (3H_A_ + 3H_B_, m), 1.25–1.42 (4H_A + B_, m), 1.42–1.62 (4H _A + B_, m), 2.96–3.05 (1H_A_ or 1H_B_, m), 3.08 (3H_A_ or 3H_B_, s), 3.10 (3H_A_ or 3H_B_, s), 3.11 (3H_A_ or 3H_B_, s), 3.14 (3H_A_ or 3H_B_, s), 3.15–3.25 (1H_A_ or 1H_B_, m), 3.87 (3H_A_ or 3H_B_, s), 3.89 (3H_A_ or 3H_B_, s), 4.02–4.16 (2H_A + B_, m), 5.24 (1H_A_, d, *J* 1.8 Hz), 5.62 (1H_B_, d, *J* 6.0 Hz), 5.86 (1H_A_, d, *J* 1.9 Hz), 6.51 (1H_B_, d, *J* 6.0 Hz), 7.55 (2H_A_ or 2H_B_, d, *J* 8.3 Hz), 7.57 (2H_A_ or 2H_B_, d, *J* 8.2 Hz), 7.98 (2H_A_ or 2H_B_, d, *J* 8.3 Hz), 8.07 (2H_A_ or 2H_B_, d, *J* 8.2 Hz). ^13^C NMR (CDCl_3_, 101 MHz) δ 13.71, 13.76, 19.91, 20.07, 29.19, 29.60, 43.65, 44.18, 44.42, 44.48, 48.01, 49.21, 52.33, 52.46, 68.50, 69.95, 83.14, 84.60, 99.66, 99.93, 128.13, 128.25, 128.57, 128.97, 136.58, 138.89, 142.50, 142.79, 158.61, 160.20, 162.80, 164.22. HRMS (ESI) *m/z* [M + H]^+^ 461.1044 (calcd for C_18_H_25_N_2_O_8_S_2_^+^, 461.1047).

*Methyl (E)-2-(1-benzyl-4-cyclohexyl-3-nitroazetidin-2-ylidene)-2-(methylsulfonyl)acetate* (**11**). Orange solid. ^1^H NMR (CDCl_3_, 300 MHz) δ 0.82–1.36 (4H, m), 1.47–1.96 (7H, m), 3.09 (3H, s), 3.70 (3H, s), 4.04 (1H, dd, *J* 4.9, 2.0 Hz), 4.36 (1H, d, *J* 15.8 Hz), 5.34 (1H, d, *J* 15.8 Hz), 5.92 (1H, d, *J* 1.9 Hz), 7.29 (2H, d, *J* 1.9 Hz), 7.27–7.46 (3H, m). ^13^C NMR (CDCl_3_, 101 MHz) δ 25.34, 25.62, 25.93, 26.38, 28.26, 38.20, 44.17, 52.22, 72.96, 80.82, 98.83, 127.76, 128.43, 129.17, 133.73, 158.80, 163.24 (two carbons accidentally isochronous). HRMS (ESI) *m/z* [M + H]^+^ 423.1581 (calcd for C_20_H_27_N_2_O_6_S^+^, 423.1584).

*Methyl (E)-2-(1-benzyl-3-nitro-4-(thiophen-2-yl)azetidin-2-ylidene)-2-(methylsulfonyl)acetate* (**15**). Whitish solid (taken up with petroleum ether). The sample analyzed at the NMR was a diastereomeric mixture (A, 43%, *cis*; B 57% *trans*). On time, A becomes the prevalent isomer. ^1^H NMR (CDCl_3_, 300 MHz) δ 3.15 (3H_B_, s), 3.16 (3H_A_, s), 3.76 (3H_B_, s), 3.83 (3H_A_, s), 4.19 (1H_B_, d, *J* 15.6 Hz), 4.33 (1H_A_, d, *J* 15.0 Hz), 5.28 (1H_B_, d, *J* 2.0 Hz), 5.46–5.57 (2H_A_ + 1H_B_, m), 6.08 (1H_B_, d, *J* 2.0 Hz), 6.40 (1H_A_, d, *J* 5.8 Hz), 7.01–7.10, 7.11–7.22, 7.28–7.38 (in all, 6H_A_ + 6H_B_, m), 7.42 (1H_A_, d, *J* 5.0 Hz), 7.49 (1H_B_, d, *J* 4.6 Hz). ^13^C NMR (CDCl_3_, 75 MHz) δ 43.58, 44.04, 51.24, 51.76, 52.21, 52.23, 63.48, 65.70, 84.52, 86.02, 99.63, 100.31, 127.82, 127.92, 127.94, 128.33, 128.56, 128.63, 128.75, 129.02, 129.04, 129.06, 129.11, 129.40, 131.78, 133.23, 133.47, 135.04, 158.56, 158.75, 162.95, 163.13. HRMS (ESI) *m/z* [M + H]^+^ 423.0683 (calcd for C_18_H_19_N_2_O_6_S_2_^+^, 423.0679).

*Methyl (E)-2-(1-(2-chlorobenzyl)-3-nitro-4-(thiophen-2-yl)azetidin-2-ylidene)-2-(methylsulfonyl) acetate* (**16**). Whitish solid. M.p. 65.1–83.4 (taken up with petroleum ether). The sample analyzed at the NMR was a diastereomeric mixture (A, 43%, *cis*; B, 57% *trans*). On time, A becomes the prevalent isomer. ^1^H NMR (CDCl_3_, 300 MHz) δ 3.16 (3H_A_ + 3H_B_, s), 3.70 (3H_B_, s), 3.78 (3H_A_, s), 4.42 (1H_B_, d, *J* 16.2 Hz), 4.68 (1H_A_, d, *J* 15.5 Hz), 5.28 (1H_B_, d, *J* 1.9 Hz), 5.32–5.44 (1H_A_ + 1H_B_, m), 5.50 (1H_A_, d, *J* 5.9 Hz), 6.07 (1H_B_, d, *J* 1.9 Hz), 6.46 (1H_A_, d, *J* 5.8 Hz), 6.98–7.38 (7H_A_ + 6H_B_, m), 7.46 (1H_B_, dd, *J* 4.5, 1.8 Hz). ^13^C NMR (CDCl_3_, 75 MHz) δ 43.53, 44.06, 49.19, 50.22, 52.16, 64.46, 66.28, 84.15, 86.15, 100.26, 100.71, 127.36, 127.40, 127.70, 127.93, 128.41, 128.73, 128.87, 129.33, 129.44, 129.75, 129.92, 129.96, 130.10, 130.61, 130.80, 131.16, 131.98, 133.34, 133.83, 135.08, 158.46, 159.56, 162.92, 162.95 (two carbons accidentally isochronous). HRMS (ESI) *m/z* [M + H]^+^ 457.0285 (calcd for C_18_H_18_ClN_2_O_6_S_2_^+^, 457.0289).

*Methyl (E)-2-(1-(3-chlorobenzyl)-3-nitro-4-(thiophen-2-yl)azetidin-2-ylidene)-2-(methylsulfonyl) acetate* (**17**). Whitish solid. M.p. 63.2–84.0 (taken up with petroleum ether). The sample analyzed at the NMR was a diastereomeric mixture (A, 43%, *cis*; B, 57% *trans*). On time, A becomes the prevalent isomer. ^1^H NMR (CDCl_3_, 300 MHz) δ 3.13 (3H_B_, s), 3.15 (3H_A_, s), 3.76 (3H_B_, s), 3.81 (3H_A_, s), 4.19 (1H_B_, d, *J* 15.7 Hz), 4.35 (1H_A_, d, *J* 15.2 Hz), 5.32 (1H_B_, d, *J* 2.1 Hz), 5.39–5.51 (1H_A_ + 1H_B_, m), 5.54 (1H_A_, d, *J* 5.8 Hz), 6.09 (1H_B_, d, *J* 2.0 Hz), 6.43 (1H_A_, d, *J* 5.8 Hz), 7.00–7.30 (6H_A_ + 6H_B_, m), 7.42 (1H_A_, dd, *J* 5.0, 1.3 Hz), 7.48–7.50 (1H_B_, m). ^13^C NMR (CDCl_3,_ 101 MHz) δ 43.77, 44.15, 50.74, 51.14, 52.46, 63.94, 66.11, 84.94, 86.10, 100.13, 100.77, 126.03, 126.71, 128.02, 128.15, 128.17, 128.64, 128.75, 128.89, 128.92, 129.11, 129.37, 129.78, 130.48, 131.62, 134.84, 134.99, 135.37, 135.75, 158.64, 158.73, 163.01, 163.26 (three carbons accidentally isochronous). HRMS (ESI) *m/z* [M + H]^+^ 457.0288 (calcd for C_18_H_18_ClN_2_O_6_S_2_^+^, 457.0289).

*Methyl (E)-2-(1-(4-chlorobenzyl)-3-nitro-4-(thiophen-2-yl)azetidin-2-ylidene)-2-(methylsulfonyl) acetate* (**18**). Whitish solid (taken up with petroleum ether). The sample analyzed at the NMR was a diastereomeric mixture (A, 42%, *cis*; B, 58% *trans*). On time, A becomes the prevalent isomer. ^1^H NMR (CDCl_3_, 300 MHz) δ 3.14 (3H_B_, s), 3.16 (3H_A_, s), 3.78 (3H_B_, s), 3.84 (3H_A_, s), 4.18 (1H_B_, d, *J* 15.6 Hz), 4.32 (1H_A_, d, *J* 15.0 Hz), 5.27 (1H_B_, d, *J* 2.0 Hz), 5.38–5.64 (2H_A_ + 1H_B_, m), 6.09 (1H_B_, d, *J* 2.0 Hz), 6.41 (1H_A_, d, *J* 5.7 Hz), 6.99–7.42 (6H_A_ + 6H_B_, m), 7.43 (1H_A_, d, *J* 5.1 Hz), 7.49 (1H_B_, d, *J* 4.4 Hz). ^13^C NMR (CDCl_3_, 75 MHz) δ 43.71, 44.13, 50.69, 51.05, 52.43, 52.46, 63.59, 65.85, 84.79, 86.07, 99.93, 100.68, 128.02, 128.13, 128.86, 129.06, 129.28, 129.37, 129.41, 129.43, 129.68, 130.08, 131.63, 131.85, 132.19, 134.44, 134.78, 134.87, 158.65, 158.80, 163.07, 163.32. HRMS (ESI) *m/z* [M + H]^+^ 457.0293 (calcd for C_18_H_18_ClN_2_O_6_S_2_^+^, 457.0289.

*Methyl (E)-2-(1-(3,4-dichlorobenzyl)-3-nitro-4-(thiophen-2-yl)azetidin-2-ylidene)-2-(methylsulfonyl) acetate* (**19**). Whitish solid. M.p. 69.8–73.7 (taken up with petroleum ether). The sample analyzed at the NMR was a diastereomeric mixture (A, 47%, *cis*; B, 53% *trans*). On time, A becomes the prevalent isomer. ^1^H NMR (CDCl_3_, 300 MHz) δ 3.13 (3H_B_, s), 3.15 (3H_A_, s), 3.78 (3H_B_, s), 3.83 (3H_A_, s), 4.19 (1H_B_, d, *J* 15.7 Hz), 4.35 (1H_A_, d, *J* 15.1 Hz), 5.32 (1H_B_, d, *J* 2.0 Hz), 5.44 (1H_A_ + 1H_B_, m,), 5.53 (1H_A_, d, *J* 5.5 Hz), 6.10 (1H_B_, d, *J* 2.0 Hz), 6.44 (1H_A_, d, *J* 5.6 Hz), 6.97–7.53 (6H_A_ + 6H_B_, m). ^13^C NMR (CDCl_3_, 75 MHz) δ 43.69, 44.05, 50.13, 50.46, 52.40, 63.87, 66.06, 84.93, 85.98, 100.30, 100.96, 127.15, 127.82, 127.95, 128.10, 128.91, 129.10, 129.33, 129.71, 129.93, 130.55, 131.02, 131.05, 131.38, 132.60, 132.91, 133.16, 133.18, 133.48, 133.93, 134.61, 158.43, 158.71, 162.93, 163.21 (two carbons accidentally isochronous). HRMS (ESI) *m/z* [M + H]^+^ 490.9898 (calcd for C_18_H_17_Cl_2_N_2_O_6_S_2_^+^, 490.9900).

*Methyl (E)-2-(1-(2-chlorobenzyl)-3-nitro-4-(p-tolyl)azetidin-2-ylidene)-2-(methylsulfonyl)acetate* (**20**). Whitish solid. M.p. 66.2–74.2 (taken up with petroleum ether). The sample analyzed at the NMR was a diastereomeric mixture (A, 45%, *cis*; B 55% *trans*). On time, A becomes the prevalent isomer. ^1^H NMR (CDCl_3_, 300 MHz) δ 2.32 (3H_A_, s), 2.38 (3H_B_, s), 3.13 (3H_A_, s), 3.15 (3H_B_, s), 3.70 (3H_B_, s), 3.82 (3H_A_, s), 4.28 (1H_B_, d, *J* 16.1 Hz), 4.57 (1H_A_, d, *J* 15.4 Hz), 4.93 (1H_B_, d, *J* 1.9 Hz), 5.21 (1H_A_, d, *J* 6.0 Hz), 5.34–5.53 (1H_A_ + 1H_B_, m), 5.94 (1H_B_, d, *J* 1.9 Hz), 6.41 (1H_A_, d, *J* 5.9 Hz), 7.05–7.35 (8H_A_ +8 H_B_, m). ^13^C NMR (CDCl_3_, 75 MHz) δ 21.30, 21.33, 43.45, 44.06, 49.26, 50.43, 52.11, 68.82, 70.95, 83.40, 85.44, 99.69, 100.07, 126.76, 127.02, 127.12, 127.30, 127.38, 129.17, 129.46, 129.68, 129.71, 129.92, 130.15, 130.26, 130.45, 130.77, 130.87, 131.22, 133.35, 133.96, 140.58, 140.99, 159.00, 160.71, 163.09, 163.14 21 (two carbons accidentally isochronous). HRMS (ESI) *m/z* [M + H]^+^ 465.0885 (calcd for C_21_H_22_ClN_2_O_6_S^+^, 465.0882).

*Methyl (E)-2-(1-(3-chlorobenzyl)-3-nitro-4-(p-tolyl)azetidin-2-ylidene)-2-(methylsulfonyl)acetate* (**21**). Whitish solid. M.p. 67.3–78.7 (taken up with petroleum ether). The sample analyzed at the NMR was a diastereomeric mixture (A, 37%, *cis*; B 63% *trans*). On time, A becomes the prevalent isomer. ^1^H NMR (CDCl_3_, 300 MHz) δ 2.35 (3H_A_ s), 2.39 (3H_B_, s), 3.13 (3H_A_ + 3H_B_, s), 3.76 (3H_B_, s), 3.85 (3H_A_, s), 4.07 (1H_B_, d, *J* 15.6 Hz), 4.25 (1H_A_, d, *J* 15.0 Hz), 4.99 (1H_B_, d, *J* 2.0 Hz), 5.25 (1H_A_, d, *J* 5.9 Hz), 5.46 (1H_B_, d, *J* 15.6 Hz), 5.55 (1H_A_, d, *J* 15.0 Hz), 5.98 (1H_B_, d, *J* 1.9 Hz), 6.40 (1H_A_, d, *J* 5.9 Hz), 7.02–7.29 (8H_A_ + 8H_B_, m). ^13^C NMR (CDCl_3_, 75 MHz) δ 21.31, 21.34, 43.57, 44.03, 50.69, 51.29, 52.26, 68.21, 70.75, 83.99, 85.25, 99.55, 100.21, 125.95, 126.31, 126.69, 127.20, 127.37, 128.10, 128.44, 128.74, 128.78, 128.84, 129.92, 130.31, 130.37, 130.41, 134.82, 134.88, 135.30, 135.67, 140.90, 141.22, 159.16, 159.66, 163.05, 163.31 (two carbons are accidentally isochronous). HRMS (ESI) *m/z* [M + H]^+^ 465.0883 (calcd for C_21_H_22_ClN_2_O_6_S^+^, 465.0882).

*Methyl (E)-2-(1-(4-chlorobenzyl)-3-nitro-4-(p-tolyl)azetidin-2-ylidene)-2-(methylsulfonyl)acetate* (**22**). Pale yellow solid. ^1^H NMR (CDCl_3_, 300 MHz) δ 2.35 (3H_A_, s), 2.40 (3H_B_, s), 3.14 (3H_A_ + 3H_B_, s), 3.78 (3H_B_, s), 3.86 (3H_A_, s), 4.05 (1H_B_, d, *J* 15.5 Hz), 4.23 (1H_A_, d, *J* 15.0 Hz), 4.93 (1H_B_, d, *J* 1.9 Hz), 5.20 (1H_A_, d, *J* 6.0 Hz), 5.50 (1H_B_, d, *J* 15.5 Hz), 5.59 (1H_A_, d, *J* 15.0 Hz), 5.97 (1H_B_, d, *J* 1.9 Hz), 6.38 (1H_A_, d, *J* 5.9 Hz), 6.85–7.45 (8H_A_ + 8H_B_, m). ^13^C NMR (CDCl_3_, 75 MHz) δ 21.32, 21.34, 43.52, 44.04, 50.61, 51.21, 52.24, 67.90, 70.47, 83.89, 85.24, 99.32, 100.04, 127.18, 127.34, 129.20, 129.28, 129.35, 129.92, 130.08, 130.36, 130.55, 131.07, 131.75, 132.11, 134.25, 134.66, 140.89, 141.19, 159.24, 159.69, 163.12, 163.35 (two carbons are accidentally isochronous). HRMS (ESI) *m/z* [M + H]^+^ 465.0880 (calcd for C_21_H_22_ClN_2_O_6_S^+^, 465.0882).

*Methyl (E)-2-(1-(3,4-dichlorobenzyl)-3-nitro-4-(p-tolyl)azetidin-2-ylidene)-2-(methylsulfonyl)acetate* (**23**). Whitish solid. M.p. 68.2–73.1 (taken up with petroleum ether). The sample analyzed at the NMR was a diastereomeric mixture (A, 37%, *cis*; B 63% *trans*). On time, A becomes the prevalent isomer. ^1^H NMR (CDCl_3_, 300 MHz) δ 2.35 (3H_A_, s), 2.40 (3H_B_, s), 3.13 (3H_B_, s), 3.14 (3H_A_, s), 3.78 (3H_B_, s), 3.85 (3H_A_, s), 4.07 (1H_B_, d, *J* 15.6 Hz), 4.26 (1H_A_, d, *J* 15.1 Hz), 4.98 (1H_B_, d, *J* 1.9 Hz), 5.25 (1H_A_, d, *J* 5.9 Hz), 5.44 (1H_B_, d, *J* 15.6 Hz), 5.52 (1H_A_, d, *J* 15.2 Hz), 5.99 (1H_B_, d, *J* 1.9 Hz), 6.41 (1H_A_, d, *J* 5.9 Hz), 6.92–7.06 (2H_A_ + 2H_B_, m), 7.07–7.31 (10H_A_ + 10H_B_, m), 7.32–7.47 (2H_A_ + 2H_B_, m). ^13^C NMR (CDCl_3_, 75 MHz) δ 21.31, 21.34, 43.59, 44.04, 50.18, 50.72, 52.33, 68.27, 70.80, 84.09, 85.21, 99.74, 100.42, 126.19, 127.20, 127.22, 127.38, 127.95, 128.67, 129.96, 130.00, 130.41, 130.46, 130.67, 130.99, 132.50, 132.89, 133.07, 133.13, 133.50, 133.95, 141.02, 141.35, 159.28, 159.59, 163.09, 163.38 (two carbons are accidentally isochronous). HRMS (ESI) *m/z* [M + H]^+^ 499.0495 (calcd for C_21_H_21_Cl_2_N_2_O_6_S^+^, 499.0492).

*Methyl (E)-2-(1-benzyl-4-(4-(methylsulfonyl)phenyl)-3-nitroazetidin-2-ylidene)-2-(methylsulfonyl) acetate* (**24**). Viscous yellow oil. ^1^H NMR (CDCl_3_, 300 MHz) δ 3.07 (3H_B_, s), 3.08 (3H _A_, s), 3.14 (3H_B_, s), 3.15 (3H_A_, s), 3.81 (3H_A_, s), 3.89 (3H_B_, s), 4.20 (1H_A_, d, *J* 15.4 Hz), 4.34 (1H_B_, d, *J* 14.9 Hz), 5.06 (1H_A_, d, *J* 1.9 Hz), 5.32 (1H_B_, d, *J* 6.2 Hz), 5.45 (1H_A_, d, *J* 15.4 Hz), 5.52 (1H_B_, d, *J* 14.9 Hz), 5.93 (1H_A_, d, *J* 1.9 Hz), 6.45 (1H_B_, d, *J* 6.2 Hz), 7.03–7.16 (4H_A + B_, m), 7.27–7.38 (6H_A + B_, m), 7.38–7.49 (4H_A + B_, m), 7.94 (2H_B_, d, *J* 8.4 Hz), 8.01 (2H_A_, d, *J* 8.3 Hz). ^13^C NMR (CDCl_3_, 101 MHz) δ 43.55, 44.02, 44.23, 44.29, 52.00, 52.32, 52.34, 52.78, 67.12, 69.20, 83.06, 84.43, 100.14, 100.59, 127.99, 128.10, 128.15, 128.44, 128.58, 128.65, 128.75, 128.83, 129.09, 129.13, 132.51, 132.66, 136.08, 138.45, 142.23, 142.51, 158.58, 159.77, 162.82, 162.89. HRMS (ESI) *m/z* [M + H]^+^ 495.0894 (calcd for C_21_H_23_N_2_O_8_S_2_^+^, 495.0890).

*Methyl (E)-2-(1-benzyl-4-(3,5-bis(trifluoromethyl)phenyl)-3-nitroazetidin-2-ylidene)-2-(methylsul-fonyl)acetate* (**25**). Yellow oil. ^1^H NMR (CDCl_3_, 300 MHz) δ 3.17 (3H_A_ or 3H_B_, s), 3.18 (3H_A_ or 3H_B_, s), 3.85 (3H_A_ or 3H_B_, s), 3.92 (3H_A_ or 3H_B_, s), 4.38 (1H_A_ or 1H_B_, d, *J* 15.2 Hz), 4.49 (1H_A_ or 1H_B_, d, *J* 14.8 Hz), 5.10 (1H_A_, d, *J* 2.0 Hz), 5.28–5.40 (2H, 1H_B_ + 1 H_A_ or 1H_B_ m), 5.44 (1H_A_ or 1H_B_, d, *J* 14.9 Hz), 5.98 (1H_A_, d, *J* 2.0 Hz), 6.48 (1H_B_, d, *J* 6.1 Hz), 7.03–7.13 (4H_A_ + 4H_B_, m), 7.26–7.36 (6H_A_ + 6H_B_, m), 7.58 (2H_A_ or 2H_B_, s), 7.64 (2H_A_ or 2H_B_, s), 7.89 (1H_A_ or 1H_B_, s), 7.94 (1H_A_ or 1H_B_, s). ^13^C NMR (CDCl_3_, 101 MHz) δ 43.73, 52.58, 53.48, 67.16, 83.19, 101.56, 122.75 (q, *J* 274.72 Hz), 124.38 (app t), 127.69, 129.08, 129.13, 129.29, 132.50, 132.51 (q, *J* 34.34 Hz), 133.32, 159.96, 162.94. HRMS (ESI) *m/z* [M + H]^+^ 553.0860 (calcd for C_22_H_19_F_6_N_2_O_6_S^+^, 553.0863).

*Methyl (E)-2-(4-(3,5-bis(trifluoromethyl)phenyl)-1-(4-chlorobenzyl)-3-nitroazetidin-2-ylidene)-2-(methylsulfonyl)acetate* (**26**). Whitish solid (taken up with petroleum ether). The sample analyzed at the NMR was a diastereomeric mixture (A, 67%, *cis*; B, 33% *trans*). ^1^H NMR (CDCl_3_, 300 MHz) δ 3.16 (3H_A_, s), 3.17 (3H_B_, s), 3.84 (3H_B_, s), 3.92 (3H_A_, s), 4.32 (1H_B_, d, *J* 15.4 Hz), 4.47 (1H_A_, d, *J* 14.9 Hz), 5.13 (1H_B_, d, *J* 2.0 Hz), 5.32–5.48 (2H_A_ + 1H_B_, m), 6.00 (1H_B_, d, *J* 2.0 Hz), 6.50 (1H_A_, d, *J* 5.9 Hz), 7.06 (2H_A_ + 2H_B_, d, *J* 8.4 Hz), 7.24–7.33 (2H_A_ + 2H_B_, m), 7.62–7.71 (2H_A_ + 2H_B_, m), 7.91 (1H_A_, s), 7.97 (1H_B_, s). ^13^C NMR (CDCl_3_, 75 MHz) δ 43.65, 44.07, 51.66, 52.47, 52.57, 67.01, 69.18, 83.24, 84.26, 101.34, 102.04, 122.53 (q, *J* 273.61 Hz), 122.62 (q, *J* 273.61 Hz), 124.53–124.68 (2C, m), 127.30, 127.58, 129.43, 129.47, 129.60, 130.17, 131.16, 131.19, 132.63 (q, *J* 33.80 Hz), 132.92, 133.37 (q, *J* 33.98 Hz), 134.96, 135.21, 135.35, 158.59, 159.58, 162.80, 162.92 (two carbons are accidentally isochronous). HRMS (ESI) *m/z* [M + H]^+^ 587.0477 (calcd for C_22_H_18_ClF_6_N_2_O_6_S^+^, 587.0473).

### 3.2. Microbiology

#### 3.2.1. Antibacterial Assay

Following a previously reported procedure [31], the minimum inhibitory concentration (MIC), i.e., the lowest concentration of the tested compound, expressed as μg/mL (γ), which prevented any visible growth of the stated bacteria, was evaluated. Briefly, MIC determinations were performed by the microdilution method, according to the Clinical laboratory Standards Institute (CLSI) guidelines with few modifications. The MIC values were obtained through duplicate experiments.

Compounds **5**–**26** were tested on the following microorganisms: Gram-positive bacteria: *S. aureus* (ATCC 29213), *S. aureus* (ATCC 6538 P), *S. aureus* (ATCC 6538), *E. faecalis* (ATCC 29212); Gram-negative bacteria: *P. aeruginosa* (ATCC 27853), *E. coli* (ATCC 25922), *K. pneumoniae* (ATCC 13883); fungi: *C. albicans* (ATCC 10231), *C. albicans* (ATCC 90028), *C. glabrata* (ATCC 15126), *C. tropicalis* (ATCC 750), *C. kefyr* (ATCC 204,093 *C. krusei* (ATCC 6258); and clinical isolates *C. albicans* 810, *C. krusei* 31A29, *C. parapsilosis* 11A13, *C. parapsilosis* 1A1 (from the Biomedical Sciences and Human Oncology Department of the University of Bari Aldo Moro, Bari, Italy).

#### 3.2.2. Evaluation of Membrane Permeability

Overnight cultures of *S. aureus* (ATCC 6538 P) and *E. faecalis* (ATCC 29212) were freshly inoculated (50 μL) in 5 mL of Mueller Hinton Broth (MHB) and incubated at 37 °C for 240 min until reaching a final optical density at 600 nm of 0.2 ± 0.3 (corresponding to 9.47 ± 0.14 log CFU/mL, on average). After centrifugation at 9500 rpm, 4 °C for 20 min, the supernatant was discarded, and the pellet was washed twice with 0.01 M potassium phosphate buffer solution (PBS, pH 7.4) before being re-suspended in the same volume of PBS. Then, 100 μL of each bacterial suspension was transferred to Eppendorf tubes containing 1 mL of PBS supplemented with **18**, **21** and **22** at concentrations equal to their corresponding MIC values or four, eight and 10-fold higher. Different concentrations of the antibiotic bacitracin (0.1, 0.4, 0.8 and 1 μg/mL) were added as positive control. Control samples were prepared similarly without treatment. Test tubes, in triplicates, were incubated at 37 °C for 1 h at 10 strokes. After incubation, the cells were harvested at 12,000 rpm for 5 min and re-suspended in PBS (200 μL) containing 10 μg/mL of crystal violet (CV). The samples were incubated for 10 min at 37 °C. The suspension was then centrifuged at 12,000 rpm for 15 min and the OD of the cell-free supernatant was measured at 570 nm. The percentage of CV uptake of all the samples was calculated using the following formula:

CV uptake (%) = ((OD570nm test)/(OD570nm CV solution)) × 100(1)

#### 3.2.3. Statistical Analysis

A one-way between-subjects ANOVA was conducted by using SPSS 20.0 (IBM SPSS, Armonk, NY, USA) to compare the effect of different concentrations (expressed in relation to respective MIC) of each assayed molecule on CV uptake percentage (%) after incubation with cells from two different target strains. Homogeneity of variances was checked using Levene’s test (*p* < 0.05). Multi-comparison analyses of means were performed with Tukey’s HSD post hoc test (*p* < 0.05).

### 3.3. Cytotoxicity Assay

This assay was performed following our previously reported procedure on HepG2 (Sigma-Aldrich, St. Louis, MO) cells [29]. Briefly, HepG2 cells were cultured in MEM with Earle’s Salts (Euroclone) supplemented with 10% (*v/v*) fatal bovine serum (Sigma-Aldrich. ST. Louis, MO), 2 mM l-glutamine (Sigma-Aldrich, St. Louis, MO), 100 mg/mL penicillin and 100 mg/mL streptomycin (Sigma-Aldrich, St. Louis, MO) at 37 °C in 5% CO_2_. The cells, grown to 70% confluence, were trypsinized using Trypsin-EDTA 1X in PBS (Aurogene) and plated in 96-well plates at a density of 10,000 cells per well in 125 μL of cell culture medium. Assays were performed 24 h after cells were seeded.

**Figure 5:**
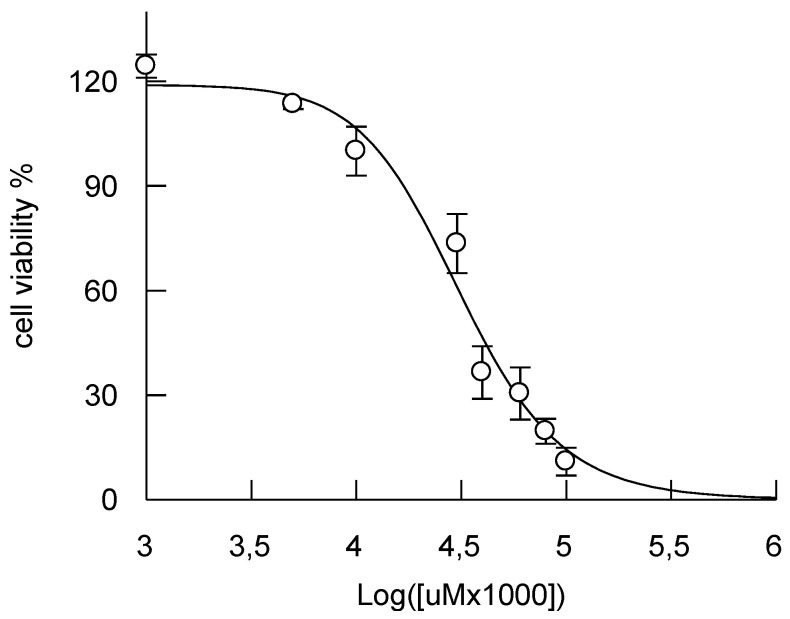
Determination of the concentration of compound **22** that caused a 50% reduction in the number of HepG2 viable cells (IC_50_).

HepG2 cell viability was assessed using a conventional 3-(4,5-dimethylthiazol-2-yl)-2,5-diphenyltetrazolium bromide (MTT) reduction assay which is based on the ability of viable cells to metabolize MTT, a water-soluble salt, by cellular oxidoreductases into a water-insoluble blue formazan product [32]. The amount of formazan produced is correlated to the viable cells (see the cited paper for details). The cells are incubated for 24 h at 37 °C in 5% CO_2_ with different concentrations of test compounds, then each well is supplemented with a solution of MTT in PBS (50 ug/mL final concentration). After 3 h of incubation, this solution is removed and 100 μL of DMSO/abs. EtOH 1:1 is added to each well. Absorbance values are measured at 570 nm using a Victor V3 plate reader (PerkinElmer) and DMSO medium is used as blank solution. Absorbance values are measured at 570 nm using a Victor V3 plate reader (PerkinElmer) and DMSO medium is used as blank solution. At least three independent experiments with six replicates (*n* 18) were carried out, and the results were averaged. The curve obtained for the determination of the concentration of compound **22** that caused a 50% reduction in the number of HepG2 viable cells (IC_50_) is shown in Figure 5.

### 3.4. Quantum Mechanical Calculations

Calculations were performed according to the procedures we previously developed [23,24]. To check the reliability of the procedure, we verify if it was able to predict the *cis*/*trans* diastereomer equilibrium found in an apolar solvent (^1^H NMR, CDCl_3_). To begin with, we focused on the relatively simple congener **5**. Briefly, the model of both *cis* and *trans* diastereomers of compound **5** were generated from the atomic fragments incorporated into Spartan’16 (Wavefunction Inc., Irvine, CA, USA) inner fragment library and assuming the suggested default starting geometries. The generated geometries were optimized by the molecular mechanics MMFF routine offered by the software [33] and then submitted to a systematic conformational distribution analysis using the default step sizes. All conformers in a window of 10 Kcal/mol above the global minimum conformer were retained. When two conformers differed by dihedral values lower than 10°, the less stable conformer was left out. Conformers were then classified according to their ab initio gas-phase energy content calculated at the RHF/6-31G* level. All conformers falling within a window of 5 kcal/mol above the global minimum were retained and submitted to RHF/6-31G* geometry optimization. After removal of redundant conformers (i.e., each conformer differing from a more stable one by less than 5° in their corresponding dihedral values), the so-obtained set of conformers underwent geometry optimization by density functional theory (DFT) implemented in Spartan’16 with B3LYP functional [34] and the 6-31G* basis set [35] in the gas phase. The optimized structures were confirmed as real minima by IR frequency calculation (DFT B3LYP/6-31G*//DFT B3LYP/6-31G*). The above geometry optimization was performed also applying the conductor-like polarizable continuum model (C-PCM; Spartan’16) to allow for apolar solvating effect consideration [36,37]. Based on the difference found between the energies of the so-obtained *cis-* and *trans*-**5** global minimum conformers (1.62 kcal/mol) a 17:1 *cis*/*trans* equilibrium ratio was found and this was in agreement with what observed through ^1^H NMR analysis. The so-obtained *cis-***5** global minimum conformer was used to obtain the corresponding model for compound **22** whose geometry was in turn optimized by DFT implemented in Spartan’20 with ῶB97X-D functional [38] and the 6-31G* basis set. For IR frequency calculation, the less costly EDF2 DFT model was used (basis set: 6-31G*).

## 4. Conclusions

When treating human infectious diseases caused by unknown microorganisms, wide spectrum antibiotics are obviously preferred. However, these powerful agents cause so-called ‘collateral damages’ (selection of resistant strains and unwanted development of colonization or infection with multidrug-resistant organisms) [39]. Furthermore, wide spectrum antimicrobials generally affect also the vitality of the microorganisms of the host microbiota. The gut microbiota is involved in the regulation of numerous physiological processes including appetite and metabolic status [40]. It has been demonstrated that an imbalance of pathogens and beneficial bacteria may have deleterious effects on the host. [41]. For example, alterations in the gut microbiota may lead to hepatocellular carcinoma [42]. Thus, when the causative pathogen is diagnosed, selective antibacterial agents should be preferred to face the infection.

Some of the nitroazetidines reported herein performed as selective and potent antibacterial agents on Gram-positive bacteria (*E. faecalis* and three strains of *S. aureus*), with the most potent congener being the *p*-chlorobenzyl, *p*-tolyl decorated nitroazetidine **22** which displayed potency close to that of norfloxacin, the reference antibiotic (minimum inhibitory concentration values 4 and 1–2 μg/mL, respectively). Since **22** is a relatively efficient agent against Gram-positive bacteria and displayed cytotoxicity on eucharyotic cells only at 4-times higher concentrations (inhibiting concentration on 50% of the cultured eukaryotic cells: 36 ± 10 μM), this nitroazetidine may be considered as a promising hit compound for the development of a new series of antibacterials selectively active on Gram-positive pathogens. The relatively concise synthetic route described herein, based as it is on easily available starting materials, could feed further structure–activity relationship studies, thus allowing for the fine investigation and optimization of the toxico-pharmacological profile.

## Figures and Tables

**Table 1:** Antibacterial activity of nitroazetidines **5–26** and norfloxacin (reference antibacterial agent) ^a^.

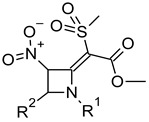							
Compound	R^1^	R^2^	*S. Aureus* ATCC 29,213 (G)	*S. Aureus* ATCC 6538 P (G +)	*S. Aureus* ATCC 6538 (G)	*E. Faecalis* ATCC 29,212 (G)	*c*Log *P*^b^
**5**	Me	*p*-tolyl	>512 (>1000)	>512 (>1000)	512 (>1000)	>512 (>1000)	0.42
**6**	Et	*p*-tolyl	>512 (>1000)	256 (696)	256 (696)	>512 (>1000)	0.75
**7**	C_6_H_11_	*p*-tolyl	>512 (>1000)	>512 (>1000)	512 (>1000)	>512 (>1000)	1.96
**8**	*n*-Bu	Ph	>512 (>1000)	>512 (>1000)	>512 (>1000)	>512 (>1000)	1.17
**9**	*n*-Bu	3,5-(CF_3_)_2_Ph	>512 (>988)	>512 (>988)	>512 (>988)	>512 (>988)	3.01
**10**	*n*-Bu	*p*-MeSO_2_Ph	256 (556)	128 (278)	128 (278)	256 (556)	0.40
**11**	Bn	C_6_H_11_	>512 (>1000)	>512 (>1000)	256 (607)	>512 (>1000)	1.91
**12**	*n*-Bu	2-thienyl	64 (165)	32 (82)	32 (82)	>512 (>1000)	−0.14
**13**	Ph	*p*-tolyl	256 (615)	256 (615)	256 (615)	256 (615)	2.49
**14**	Bn	*p*-tolyl	32 (74)	16 (37)	16 (37)	>512 (>1000)	2.10
**15**	Bn	2-thienyl	16 (38)	16 (38)	16 (38)	32 (76)	0.35
**16**	*o*-ClBn	2-thienyl	64 (140)	32 (70)	128 (280)	32 (70)	0.91
**17**	*m*-ClBn	2-thienyl	32 (70)	16 (35)	128 (280)	64 (140)	0.91
**18**	*p*-ClBn	2-thienyl	16 (35)	8 (17.5)	16 (35)	8 (17.5)	0.91
**19**	3,4-Cl_2_Bn	2-thienyl	8 (16.2)	8 (16.2)	16 (32)	16 (32)	1.47
**20**	*o*-ClBn	*p*-tolyl	32 (69)	16 (34)	128 (276)	64 (138)	2.71
**21**	*m*-ClBn	*p*-tolyl	8 (17.2)	8 (17.2)	8 (17.2)	16 (34)	2.71
**22**	*p*-ClBn	*p*-tolyl	4 (8.6)	4 (8.6)	4 (8.6)	4 (8.6)	2.71
**23**	3,4-Cl_2_Bn	*p*-tolyl	8 (16.0)	8 (16.0)	16 (32)	32 (64)	3.26
**24**	Bn	*p*-MeSO_2_Ph	256 (518)	128 (259)	256 (518)	256 (518)	0.89
**25**	Bn	3,5-(CF_3_)_2_Ph	64 (116)	8 (14.5)	64 (116)	128 (232)	3.50
**26**	*p*-ClBn	3,5-(CF_3_)_2_Ph	>512 (>1000)	>512 (>1000)	>512 (>1000)	>512 (>1000)	4.06
**norfloxacin**	*-*	-	1 (3.1)	0.50 (1.57)	0.50 (1.57)	2 (6.2)	0.82

^a^ Minimum inhibitory concentration (MIC), that is, the lowest concentration, expressed as μg/mL (γ) and μmol/L (in parentheses), that prevented any visible growth of the stated bacteria; when the MIC value was higher than 512 γ, the compound was considered to be ‘inactive’; compounds **5**–**26** displayed no activity (MIC > 512 μg/mL) on the following Gram-negative bacteria: *P. aeruginosa* (ATCC 27853), *E. coli* (ATCC 25922), and *K. pneumoniae* (ATCC 13883); the stated compounds displayed no activity (MIC > 512 μg/mL) on the following fungi: *C. albicans* (ATCC 10231), *C. albicans* (ATCC 90028), *C. glabrata* (ATCC 15126), *C. tropicalis* (ATCC 750, *C. kefyr* (ATCC 204093), *C. krusei* (ATCC 6258) and clinical isolates *C. albicans* A18, *C. albicans* 810, *C. krusei* 31A29, *C. parapsilosis* 11A13, *C. parapsilosis* 1A1 (from the Biomedical Sciences and Human Oncology Department of the University of Bari Aldo Moro); ^b^ estimated according to the method of Ghose, Pritchett and Crippen [28], implemented in Spartan ‘16 (Wavefunction, Irvine, CA, USA).

**Table 2:** Comparison of the Main Stereoelectronic Features of the Azetidine Rings in Penicillin G and 2-Nitroazetidine 22 as Obtained through Quantum-Mechanical Calculations (DFT ῶB97X-D/6-31G*//DFT ῶB97X-D/6-31G*, Gaseous Phase).

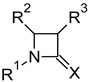			
	**Penicillin G ^a^**	**Compd 22 ^b^**	Reported qualitative relationship between β-lactam stereoelectronic features and antibacterial activity [ref1]; **↑**: improves, **↓**: lowers
C=X bond length (Å)	1.20	1.37	short **↑**; long **↓**
C-N bond length (Å)	1.40	1.35	long **↑**; short **↓**
C=X bond order			high **↑**; low **↓**
Löwdin	2.22	1.55	
Mulliken	1.92	1.53	
C=X stretching frequency (cm^−1^) ^c^	1838	1624	high **↑**; low **↓**
net atomic charge on X (units of electrons)			less negative **↑**; more negative **↓**
electrostatic	−0.45	−0.67	
Mulliken	−0.46	−0.40	
natural	−0.56	−0.57	
Sum of bond angles at nitrogen (°)	332	359	<< 360 **↑**; < 360 **↓**
Woodward *h* value ^d^	0.46	0.19	high **↑**; low **↓**
electrophilicity index (eV) ^e^	0.90	1.05	high **↑**; low **↓**

^a^ X = O; R^1^, R^2^ = CH(CO_2_H)C(CH_3_)_2_S; R^3^ = BnC(O)NH. ^b^ X = quaternary C(sp^2^); R^1^ = *p*-ClBn; R^2^ = *p*-tolyl; R^3^ = NO_2_. ^c^ DFT EDF2/6-31G*//DFT *ῶ*B97X-D/6-31G*, gaseous phase; the so-calculated IR frequencies are typically overestimated by 5%.. ^d^ distance of the nitrogen from the plane defined by its three substituent atoms. ^e^ this descriptor (*ῶ*) is related to the capacity of an electrophile to promote a soft (covalent) reaction [*ῶ* = (*I* + *A*)^2^/8(*I* − *A*) = (*E*_HOMO_ + *E*_LUMO_)^2^/8(*E*_HOMO_ − *E*_LUMO_) [29].

## Data Availability

Not applicable.

## Sample Availability

Samples of the compounds are available from the authors.
